# Ionic Liquids: Just Molten Salts After All?

**DOI:** 10.3390/molecules14072521

**Published:** 2009-07-13

**Authors:** Hon Man Yau, Si Jia Chan, Stephen R. D. George, James M. Hook, Anna K. Croft, Jason B. Harper

**Affiliations:** 1 School of Chemistry, University of New South Wales, Sydney, NSW, 2052, Australia; 2 Analytical Centre, University of New South Wales, Sydney, NSW, 2052, Australia; 3 School of Chemistry, University of Wales Bangor, Bangor, Gwynedd, LL57 2UW, UK

**Keywords:** ionic liquid, molten salt, reaction outcome, electrostatic interactions

## Abstract

While there has been much effort in recent years to characterise ionic liquids in terms of parameters that are well described for molecular solvents, using these to explain reaction outcomes remains problematic. Herein we propose that many reaction outcomes in ionic liquids may be explained by considering the electrostatic interactions present in the solution; that is, by recognising that ionic liquids are salts. This is supported by evidence in the literature, along with studies presented here.

## 1. Introduction

Ionic liquids have been touted as potential alternatives to volatile organic solvents. [1] Arbitrarily defined as having a melting point of less than 100 ºC [2], they are typically based on bulky organic cations, generally containing either cationic nitrogen or phosphorus centres, with the charge balanced by one or more of a variety of organic and inorganic anions [3,4,5,6,7]. Whilst liquid, the electrostatic interactions between the component ions result in many of the properties widely associated with ionic liquids, particularly their negligible vapour pressure [3,8]. Modification of either of the ionic components alters the physical properties [6,7,8,9,10,11], particularly miscibility with other liquids [6,7], allowing them to be ‘tuned’ to a given process and thus utilised as versatile ‘designer solvents’ [12,13,14,15,16].

A significant hurdle in the widespread application of ionic liquids is the effect that they have on reaction outcomes. When a reaction is carried out in an ionic liquid, differences in the rates and selectivities of the process are often observed when compared to the corresponding reaction in molecular solvents [17]. The variation in outcomes is elegantly demonstrated through treatment of toluene with nitric acid in a range of ionic liquids [18]. While halogenation, rather than nitration can, be readily rationalised in the halide based salts, the change in rate and selectivity observed in a bis(trifluoromethanesulfonyl)imide ionic liquid and the oxidation, rather than nitration, observed in a mesylate ionic liquid are not so easily explained.

At this stage there are limited reports detailing the origins of such changes [17], which is in marked contrast to the extensive understanding of the effect on reaction outcome upon changing from one molecular solvent to another [19]. In addition, these reports focus on using traditional solvent parameters to describe ionic liquids and, even when limited to considering Diels-Alder processes in ionic liquids with hydrogen-bonding cations [20,21,22,23,24], the methods give poor correlations, particularly for rates [24]. Further, the contributing factors vary between different reactions. At the extreme case, the contributing properties are observed to be markedly different even when the dienophile is changed in a Diels-Alder reaction [24]. This means that different reactions need to be considered in different ways, making for a very non-unified or *ad hoc* approach to explaining reaction outcomes.

Here we propose that an alternative way to understand the effects of moving to ionic liquid solvents is to consider ionic liquids as originally described in the literature; as molten salts. Being made up of ions, the principle interactions in such a solvent are electrostatic and the interactions between the component ions are typically much larger than between the ions and solute molecules. While we have used such an argument previously to describe the anomalously high solubility of benzenes in ionic liquids [25,26] and others, particularly Kobrak *et al*. [27,28,29,30], have considered this to describe the physical properties of ionic liquids, here we extend this idea as a method of rationalising reaction outcomes.

**Scheme 1:**
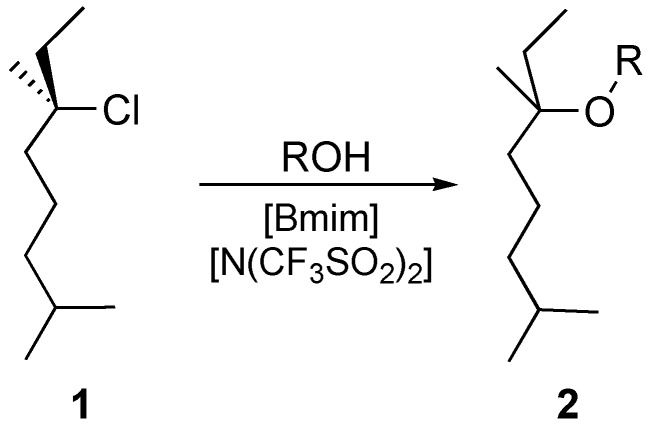
Nucleophilic substitution of the chloride **1** to give the ether **2**, carried out in a mixture of alcohol and ionic liquid.

Some of our earlier work [31,32] has focused on the effect of ionic liquids on the unimolecular substitution process outlined in Scheme 1. Particularly, the effect of the ionic liquid 1-butyl-3-methylimidazolium ([Bmim]^+^) bis(trifluoromethanesulfonyl)imide ([N(CF_3_SO_2_)_2_]^-^) on this chloride solvolysis was shown to be a decrease in both the enthalpy and entropy of activation.[32] This was rationalised through a stabilisation of the incipient charges in the transition state, with an increase in the ordering of the ionic components of the solution required to achieve this. A key experiment included in that work was an example where an 'ordinary' salt, in this case lithium bis(trifluoromethanesulfonyl)imide was added. The effects on the reaction were observed to be (qualitatively) similar to those obtained with the ionic liquid. This 'salt effect' was used to rationalise an initial rate increase seen on addition of a small amount of the ionic liquid. However, it also introduced the possibility that ionic liquids could be considered simply as molten salts in terms of explaining their observed effects. As such, it serves as a starting point for the work described here.

## 2. Results and Discussion

Clearly, given the above observations, it is of interest to determine whether the effects of ionic liquids on reaction outcomes could be mimicked by salts that are not liquids at room temperature. Since we have an interest in understanding these reaction processes at the molecular (or ionic!) level, we are particularly interested in the effect of salts on the kinetics and selectivities of such processes and have focused on systems for which we have some background. In addition, we discuss a prominent example from the ionic liquid literature where treating the ionic liquid simply as a salt may provide some insight along with highlighting the potential of molecular dynamics simulations for these systems and how such simulations fit well into this argument.

### 2.1. Bimolecular substitution of a benzyl bromide

**Scheme 2:**
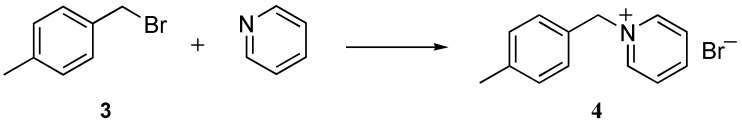
Menschutkin reaction of the benzyl bromide **3** with pyridine.

The Menschutkin reaction [33] of pyridine and the benzyl bromide **3** (Scheme 2) was considered as it is related to the unimolecular process described above, however, the extent of charge development is less than for the unimolecular process. The reaction was carried out in acetonitrile, acetonitrile containing lithium bis(trifluoromethanesulfonyl)imide (at *χ* = 0.10) and in [Bmim][N(CF_3_SO_2_)_2_] (at *χ* = 0.87). Reaction progress was monitored using ^1^H-NMR spectrosopy over a range of temperatures and the activation parameters (Table 1) were calculated using the Eyring equation.[34].

**Table 1:** Activation parameters for the reaction outlined in Scheme 2 in the solvent system specified. Uncertainties quoted are standard deviations.

Solvent	ΔH^‡^ (kJ mol^-1^)	ΔS^‡ ^(J K^-1^ mol^-1^)
Acetonitrile	43.2 ± 1.1	-219 ± 4
Acetonitrile containing Li[N(CF_3_SO_2_)_2_]	57.1 ± 0.5	-191 ± 2
[Bmim][N(CF_3_SO_2_)_2_]	48.8 ± 0.9	-193 ± 3

The effect of the ionic liquid, in this case, is opposite to that observed previously [32] with an increase in both the enthalpy and entropy of reaction. While the origin of the changes in the activation parameters on addition of an ionic liquid are discussed in detail elsewhere [35], these results imply an increase in stabilisation of, and ordering about, the starting material relative to the transition state on going from acetonitrile to the systems containing ions. Importantly for the argument presented here, the effect of the corresponding lithium salt was the same, with only the magnitude of the change in activation enthalpy being larger. This difference is presumably as a result of the more charge-dense lithium cation. That is, this case represents a further example where the effect of an ionic liquid is comparable to an 'ordinary' salt.

### 2.2. Hydrolysis of benzaldehyde dimethyl acetal (**5**)

**Scheme 3:**
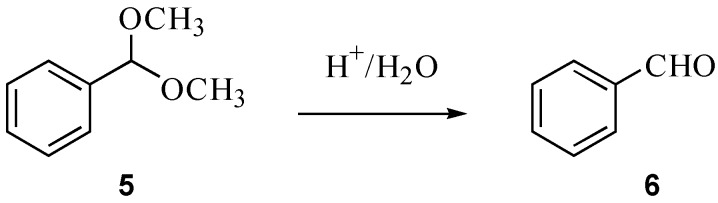
Acid-catalysed hydrolysis of benzaldehyde dimethyl acetal **5**.

A further reaction we have considered in ionic liquids is the hydrolysis of the acetal **5** to the corresponding aldehyde **6** (Scheme 3). This reaction was considered as, along with being a markedly different reaction type to the substitutions described above, it also requires a water-miscible ionic liquid, in contrast to the systems already studied. As such, the reaction was carried out in mixtures of acetonitrile/water (10:1), acetonitrile/water containing sodium chloride (at *χ* = 0.07) and in acetonitrile/ water containing [Bmim]Cl (at *χ* = 0.21). Using ^17^O-NMR spectroscopy, the reaction progress was followed over a range of temperatures and the activation parameters (Table 2) were calculated using the Eyring equation [34]. [Bmim]Cl, while often used simply as a precursor to other ionic liquids, is also an ionic liquid itself and the mole fraction used here corresponds to *ca*. 50% by weight.

It is important to note that the reaction does not have a single elementary step; a proton transfer equilibrium exists prior to the rate determining step. As such, changing the temperature and the solvent may affect the position of this equilibrium. The linearity of the Eyring plots obtained suggests otherwise, in this case. Further, a systematic error is introduced in the observed entropies of activation. No attempt to determine this error is made, as it is difficult to ascertain and the entropies of activation can only be reported taking note of this deviation (for details, see Supplementary Information). Given that it is the changes in these parameters, not their absolute values, that are of interest, this systematic error does not affect the argument presented.

**Table 2:** Activation parameters for the reaction outlined in Scheme 3 in the solvent systems specified. Uncertainties quoted are standard deviations.

Solvent	ΔH^‡^ (kJ mol^-1^)	ΔS^‡ ^(J K^-1^ mol^-1^)
Acetonitrile/water (10:1)	136 ± 5	139 ± 17
Acetonitrile/water containing NaCl	110 ± 3	44 ± 10
Acetonitrile/water containing [Bmim]Cl	71 ± 4	-84 ± 13

Once again, the effect on the activation parameters of replacing sodium chloride with the ionic liquid [Bmim]Cl is the same, leading to a reduction in both the enthalpy and entropy of activation relative to the case when both were absent. This implies that the charge separation involved in the rate determining step is favoured, presumably by the more 'ionic' environment, but that such stabilisation requires significant ordering of the components of the solution about the transition state, resulting in a decrease in the entropy of activation (it is worth highlighting that the effect on the activation parameters are the reverse of that observed in Section 2.2; this is discussed elsewhere [35]). The greater effect in the ionic liquid case can be attributed to the larger amount of [Bmim]Cl that can be dissolved in the reaction mixture when compared to sodium chloride, though the effect remains the same as the 'typical' salt.

### 2.3. A nitrile oxide cycloaddition

**Scheme 4:**
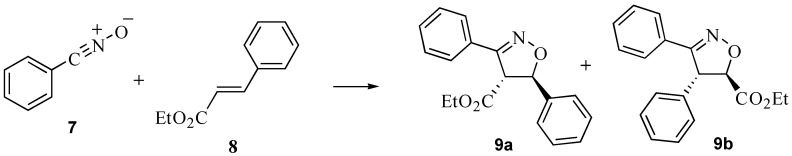
Nitrile oxide cycloaddition of benzonitrile oxide **7** to ethyl *trans*-cinnamate **8** to give the isoxazoles **9a** and **9b**.

In addition to considering the effects of ionic liquids on the kinetic parameters of organic processes, we have examined the effect of these solvents on the regioselectivity of nitrile oxide cycloadditions, such as that shown in Scheme 4 [36]. These systems have been shown to be versatile synthetic intermediates due to the high degree of functionality (for a detailed discussion, see [37]). However, for our purposes it is the mixture of regioisomers produced, which is dependent on both steric and electronic effects, which is of interest. We have shown that steric contributors to the regioselectivity are more significant in ionic liquids.[36] Whether 'ordinary' salts have the same effect was examined by considering the reaction between the nitrile oxide **7** and the cinnamate **8** in acetonitrile, acetonitrile containing lithium bis(trifluoromethanesulfonyl)imide (at *χ* = *ca.* 0.32), acetonitrile containing tetrabutylammonium tetrafluoroborate (at *χ* = *ca.* 0.32) and in [Bmim][N(CF_3_SO_2_)_2_] (at *χ* = *ca.* 0.9). (The ionic liquid is representative of one of a series used; for the others see [36].) The ratio of the isoxazoles **9a** and **9b** produced was determined using ^1^H-NMR spectroscopy (Table 3). 

**Table 3:** Ratios of the isoxazoles **9a** and **9b** produced in the reaction outlined in Scheme 4 in the solvent systems specified and extents of conversion. Uncertainties quoted are standard deviations.

Solvent	9a : 9b (% conversion)
Acetonitrile	(6.5 ± 0.4) : 1 (44%)[36]
Acetonitrile containing Li[N(CF_3_SO_2_)_2_]	(4.6 ± 0.3) : 1 (15%)
Acetonitrile containing [NBu_4_]BF_4_	(7.5 ± 0.4) : 1 (30%)
[Bmim][N(CF_3_SO_2_)_2_]	(15.2 ± 0.8) : 1 (84%)[36]

As described previously [36], the effect of ionic liquids on nitrile oxide cycloadditions was to change the ratio of the isoxazoles produced in favour of the less sterically hindered isomer. This is seen in the above table with the ratio of the isoxazoles **9a**:**9b** increasing on moving to the ionic liquid. The addition of salts to this reaction is more complicated than for the substitution reactions described above as a result of the oxygen atoms in the reagents. In the case of the lithium salt, a reversal of regioselectivity was observed along with a decrease in the extent of reaction. This is consistent with the reported effects of addition of lithium ions to nitrile oxide cycloaddition reactions in which both the dipolarophile and the nitrile oxide can coordinate to the lithium; the resulting chelation favours the isomer **9b** [38]. When the lithium salt is replaced by a tetrabutylammonium salt (a range of salts, each with a non-coordinating cation, was considered, however, many qualify as ionic liquids; this salt has a relatively high melting point of 155-161°C [39]) a small but measurable increase in the proportion of the less sterically hindered isomer **9a** is observed with a comparable extent of conversion. That is, once again the outcome of the reaction when ionic liquid is used might be considered an extension of that observed when an 'ordinary' salt is added to the reaction mixture with the greater effect accounted for by the larger mole fraction of ionic liquid present (the limitations of the potential effects of such salts are demonstrated here by the fact that the saturated ammonium salt solution used is only *χ* = *ca.* 0.32 in the salt). Note that given the observed effect of ionic liquids was attributed to the internal pressure of the ionic liquid system, it is perhaps not surprising that the addition of salts to molecular solvents has been shown to increase the internal pressure of the mixture [36] (for a discussion on the increased extent of conversion in the ionic liquid see previous work [36]).

### 2.4. Results present in the literature

As far as the authors are aware, there are no examples in the literature where the effect of a molten and a solid salt on the same reaction are compared directly. While it might be of interest to examine a range of 'unexplained' outcomes in terms of this novel argument, this is beyond the scope of this article. Rather, it is worth considering one of the most studied processes in ionic liquids, that being the Diels-Alder reaction [40], on which information on the effect of a molten and a solid salt have been reported separately.

This reaction is a particularly appropriate case to study is that this reaction has seen much investigation of the effect of dissolved salts on the outcome [41], with the use of 5 M lithium perchlorate in diethyl ether (LPDE) being particularly studied. LPDE has been demonstrated to dramatically increase the rate and stereoselectivity, with an increase in the *endo*:*exo* ratio being observed [42]. This was used to great effect in the synthesis of cantharadin, with reaction in LPDE being able to be completed under ambient conditions [42], whereas previous syntheses required ultrahigh pressure to be applied to the reaction mixture [43]. From these observations, it has been suggested that the effect of LPDE is to act as a medium with high internal pressure [44], with the effects on the Diels-Alder process being consistent with its negative volume of activation [45,46,47]. While the Lewis acidity of the lithium cation cannot be overlooked and may catalyse the cycloaddition, such acidity is moderated by the solvent [48]. The relative importance of each of these contributing factors (internal pressure and Lewis acid catalysis) is the subject of some debate and is likely to vary with the reaction considered, both factors contribute to the change in reaction outcomes of Diels-Alder processes in LPDE [49].

When ionic liquids are used as solvents for Diels-Alder reactions, an increase in the rate and the *endo*:*exo* selectivity is generally observed [50,51,52,53,54]. While, as described in the introduction, correlating these with reaction outcomes remains problematic [20,21,22,23,24] the effect of the ionic liquid is qualitatively the same as for the ‘ordinary salt’ system, LPDE. In addition, the hydrogen bonding ability of the cation was shown to be an important parameter [22,24], which is consistent with the ionic liquid acting as a Lewis acid. The influence of this on reaction outcomes has been shown to vary with the reagents considered [24] and there are some systems where hydrogen bonding is not possible, and hence the Lewis acidity of the ionic liquid greatly decreased, but the outcomes observed are similar (for example, pyridinium [53]and phosphonium [54] based ionic liquids). The factors contributing to reaction outcomes in LPDE suggest that the internal pressure of the medium, shown to be larger than for molecular solvents [55], may also be significant in this case.

This simple case study demonstrates two key points. Firstly, it is a further case where the ionic liquid can be considered to be acting in the same way as an ‘ordinary’ salt as the outcomes observed are qualitatively similar. Secondly, it demonstrates a case where an understanding of the effect of the addition of a simple salt can be used as a starting point to explain the effect of using an ionic liquid solvent.

### 2.5. Comparison with molecular dynamics simulations

It is important to note that, in one respect, treating ionic liquids in terms of molten salts to explain properties is not new. Both we [25,26,56] and many others [57] have used molecular dynamics (MD) simulations to explain the physical properties of ionic liquids, along with interactions between the ionic liquid and dissolved solutes, and the application of computational modeling to these problems is increasing [58]. In MD simulations, components of the ionic liquid are described by the use of forcefields, which contain parameters relating to the motion of bonded atoms (bending and torsions) and to non-bonded interactions (electrostatic and dispersion interactions). Several excellent forcefields exist with broad coverage for ionic liquids, for example those based on the OPLS/AMBER model [59,60,61,62,63], and information from *ab initio* studies, and particularly those incorporating dispersion interactions, is helping to further refine and improve the ionic liquid forcefield descriptions [64,65,66,67,68,69]. It should be noted, however, that the bulk of properties and interactions reflect the electrostatic contributions,[70] particularly for small cations, *i*.*e*. those with short to medium alkyl chains. Overall, these simulations can accurately describe physical and solubility properties of ionic liquid solutions and suggests that MD simulation is an appropriate way of considering such systems.

Perhaps surprisingly, given the excellent consistency with experimental data, there has been little reported use of molecular dynamics simulations to account for the effects that ionic liquids have on reaction outcomes. Partly this may be attributed to the difficulty of simulating reactive intermediates using classical MD and the computational intensity required for modeling even small timespans and molecular ensembles with *ab initio* MD methods. In terms of reactivity, simple electron transfer processes have been examined using the former [71] and proton transfers with the latter [72]. We have instead used atomistic classical MD simulations to demonstrate the increased ordering about a fixed intermediate (and by implication, about the transition state leading to it) to describe a model system for solvolysis reactions, outlined in Scheme 1 [32]. Of particular note are the significant interactions between the IL cation and the incipient charge on the chlorine atom, reflecting the importance of the electrostatic contributions.

The ability of such simulations to elegantly describe changes in reaction entropy and enthalpy on the molecular level opens up a number of possibilities. Initially, a reaction whose outcome is known in ionic liquids can be rationalised using molecular dynamics simulations, providing a microscopic viewpoint that can go hand-in-hand with the macroscopic view of the effect of the ionic liquid as a salt. Perhaps more excitingly, such an understanding (both at the microscopic and macroscopic levels) might be gained *prior* to studying the reaction experimentally. That is, this methodology can be used as a predictive tool and screening mechanism, catalysing rapid developments in the IL field.

## 3. Experimental Section

### 3.1. General

The starting materials **3**, **5** and **8** were commercially available, whilst phenylchloroaldoxime was prepared from commercially available starting materials using literature methods [73]. [Bmim][N(CF_3_SO_2_)_2_] was prepared from [Bmim]Cl, which was in turn prepared by literature methods from commercially available starting materials, and both were dried to constant weight over phosphorus pentoxide [7]. Water, where used, refers to deionised water. Pyridine was purified using a literature method [74] . Other reagents were of analytical grade and were used as received.

### 3.2. Bimolecular substitution of benzyl bromide

Kinetic analyses of the Menschutkin reaction of the benzyl bromide **3** with pyridine were carried out in solutions containing the bromide **3** (*ca*. 0.05 mol L^-1^) and pyridine (*ca*. 5-20 equiv.) in either acetonitrile, acetonitrile (5.91 g, 0.144 mol) containing lithium bis(trifluoromethanesulfonyl)imide (4.96 g, 17.3 mmol, 1.73 mol.L^-1^) or [Bmim][N(CF_3_SO_2_)_2_] at temperatures between 278 K and 312 K. In each case the reaction was followed using ^1^H-NMR spectroscopy until more than 95% of the starting material **3** was consumed. Spectra were taken at regular intervals during the reaction and at least twenty spectra were obtained for each kinetic run. The extent of reaction was deduced through integration of the signal corresponding to the benzyl protons in the starting material **3** (*ca*. δ 4.5) and the products **4** (*ca*. δ 5.8). From this information, the pseudo-first order rate constants for the reaction under these conditions was calculated and, consequently, from the concentration dependence of these values, the second order rate constants at each temperature. The activation parameters were then determined using the bimolecular Eyring equation [34].

### 3.3. Hydrolysis of benzaldehyde dimethyl acetal (**5**)

Benzaldehyde dimethyl acetal (**5**, 0.6 mL), water (0.3 mL) and pyridinium *p*-toluenesulfonate (1.5 mg) were added to acetonitrile (3 mL). The reaction mixture was monitored using ^17^O-NMR spectroscopy, specifically the disappearance of the signal corresponding to compound **5** at *δ*_O_ 18, at temperatures between 288 K and 308 K. From these, the first order rate constants at each temperature were calculated and the activation parameters determined using the unimolecular Eyring equation [34]. The process was repeated for mixtures containing the acetal **5** (0.2 mL), ^17^O-enriched water (0.1 mL; the conditions of the experiment necessitated using 0.02% ^17^O enriched water to ensure sufficient signal strength given the time constraints of the experiment), pyridinium *p*-toluenesulfonate (0.5 mg), [Bmim]Cl (0.45 g) and acetonitrile (0.5 mL) over the temperature range 288 K to 308 K. It was also repeated for mixtures containing the acetal **5** (0.2 mL), ^17^O-enriched water (0.1 mL), pyridinium *p*-toluenesulfonate (0.5 mg) and acetonitrile (1 mL) which had been saturated with sodium chloride (*ca*. 45 mg, *χ* = 0.07) over the temperature range 308 K – 328 K. In each of these latter cases, the reaction was followed by observing the appearance of the signal corresponding to the aldehyde **6** at *δ*_O_ 550.

### 3.4. A nitrile oxide cycloaddition

The reactions were carried out as described previously [36]. Briefly, the nitrile oxide **7** was produced *in situ* from the corresponding chloroaldoxime [73], through the addition of triethylamine (50 μL) to a solution of the chloroaldoxime (25 mg), the cinnamate **8** (52 mg) and either lithium bis(trifluoromethanesulfonyl)imide (5.14 g) or tetrabutylammonium tetrafluoroborate (5.85 g) in acetonitrile (2 mL) over 24 h. The regioisomeric ratio of the isoxazoles **9a** and **9b** produced and the extent of reaction was determined using ^1^H-NMR spectroscopy, specifically the integrations of the signals corresponding to the C-4 and C-5 protons of the products **9a** and **9b** and the alkene protons of the residual starting material **8** [36]. The extent of conversion of the cinnamate **8** in these cases was *ca*. 15% in the case of the lithium salt addition, 30% in the case of the tetrabutylammonium salt addition.

## 4. Conclusions

The above results demonstrate that, for a range of reaction types, the effect of ionic liquids parallels that of more 'typical' salts, though care must be taken to recognise that the components of ionic liquids tend to be comparatively non-coordinating. This is not to suggest that ionic liquids can be replaced by these salts. The benefits of ionic liquids, particularly the potential to replace volatile organic solvents and to be highly soluble in such solvents when they are required, remain. However, in terms of understanding the effects that ionic liquids have on reaction outcomes, this overview demonstrates that the starting point for proper comprehension of IL interactions is their charged nature. It is clear that, in these systems, electrostatic interactions are principal and have important application in predicting the effect of changing reaction solvent from a molecular solvent to an ionic liquid.
